# ChatGPT vs Google for Queries Related to Dementia and Other Cognitive Decline: Comparison of Results

**DOI:** 10.2196/48966

**Published:** 2023-07-25

**Authors:** Vagelis Hristidis, Nicole Ruggiano, Ellen L Brown, Sai Rithesh Reddy Ganta, Selena Stewart

**Affiliations:** 1 Department of Computer Science and Engineering University of California, Riverside Riverside, CA United States; 2 School of Social Work University of Alabama Tuscaloosa, AL United States; 3 Nicole Wertheim College of Nursing and Health Sciences Florida International University Miami, FL United States

**Keywords:** chatbots, large language models, ChatGPT, web search, language model, Google, aging, cognitive, cognition, dementia, gerontology, geriatric, geriatrics, query, queries, information seeking, search

## Abstract

**Background:**

People living with dementia or other cognitive decline and their caregivers (PLWD) increasingly rely on the web to find information about their condition and available resources and services. The recent advancements in large language models (LLMs), such as ChatGPT, provide a new alternative to the more traditional web search engines, such as Google.

**Objective:**

This study compared the quality of the results of ChatGPT and Google for a collection of PLWD-related queries.

**Methods:**

A set of 30 informational and 30 service delivery (transactional) PLWD-related queries were selected and submitted to both Google and ChatGPT. Three domain experts assessed the results for their currency of information, reliability of the source, objectivity, relevance to the query, and similarity of their response. The readability of the results was also analyzed. Interrater reliability coefficients were calculated for all outcomes.

**Results:**

Google had superior currency and higher reliability. ChatGPT results were evaluated as more objective. ChatGPT had a significantly higher response relevance, while Google often drew upon sources that were referral services for dementia care or service providers themselves. The readability was low for both platforms, especially for ChatGPT (mean grade level 12.17, SD 1.94) compared to Google (mean grade level 9.86, SD 3.47). The similarity between the content of ChatGPT and Google responses was rated as high for 13 (21.7%) responses, medium for 16 (26.7%) responses, and low for 31 (51.6%) responses.

**Conclusions:**

Both Google and ChatGPT have strengths and weaknesses. ChatGPT rarely includes the source of a result. Google more often provides a date for and a known reliable source of the response compared to ChatGPT, whereas ChatGPT supplies more relevant responses to queries. The results of ChatGPT may be out of date and often do not specify a validity time stamp. Google sometimes returns results based on commercial entities. The readability scores for both indicate that responses are often not appropriate for persons with low health literacy skills. In the future, the addition of both the source and the date of health-related information and availability in other languages may increase the value of these platforms for both nonmedical and medical professionals.

## Introduction

### Background

People living with dementia or other cognitive decline and their caregivers (collectively referred to as PLWD in this paper) often find it challenging to obtain the right information about their health, given the wide range of conditions, progression levels, symptoms, and side effects associated with dementia or other cognitive decline [1,2]. These problems are exacerbated due to the shortage of expert providers and the limited access to them, especially in rural or low-income settings [3,4]. PLWD have been using the web to find answers, often starting from a web search engine and then following relevant hyperlinks [3,4]. This is challenging, given the information overload on the web, the reliability of sources, the health literacy level of some content, and the skill required to locate the right answer [5]. Another factor that makes such query tools challenging is the wide range of education levels of PLWD.

Recent advances in deep learning, which is a type of machine learning based on artificial neural networks, have given rise to several conversational artificial intelligence (AI) platforms (chatbots). In our recent survey on chatbots for PLWD, we found that these systems have generally limited scope based on the information programmed into them by their creators [6]. They also have limited natural language–understanding capabilities. A new promising chatbot platform, ChatGPT, which was introduced in late 2022, is trained on huge amounts of data and has superior natural language–understanding technology (see the *Review of Query Tools* section for details). ChatGPT has been shown to be able to answer complex questions on a wide range of topics and even change the style of the responses based on the user (eg, change the literacy level, make the answer funny, etc).

PLWD have a wide range of needs for which they rely on the web for answers. A seminal paper on web searching identified 3 types of search needs of web users: informational, transactional, and navigational [7]. Informational queries aim to acquire some information assumed to be present on 1 or more web pages (eg, “Is there a cure for dementia?”). Transactional queries try to perform an activity (eg, “Find good home care in Riverside, California.”). In navigational queries, users look for the web address of an organization (eg, “WebMD”). We did not consider navigational queries in our study as their answers are usually trivial and they are not common in conversational settings.

**Figure 1:**
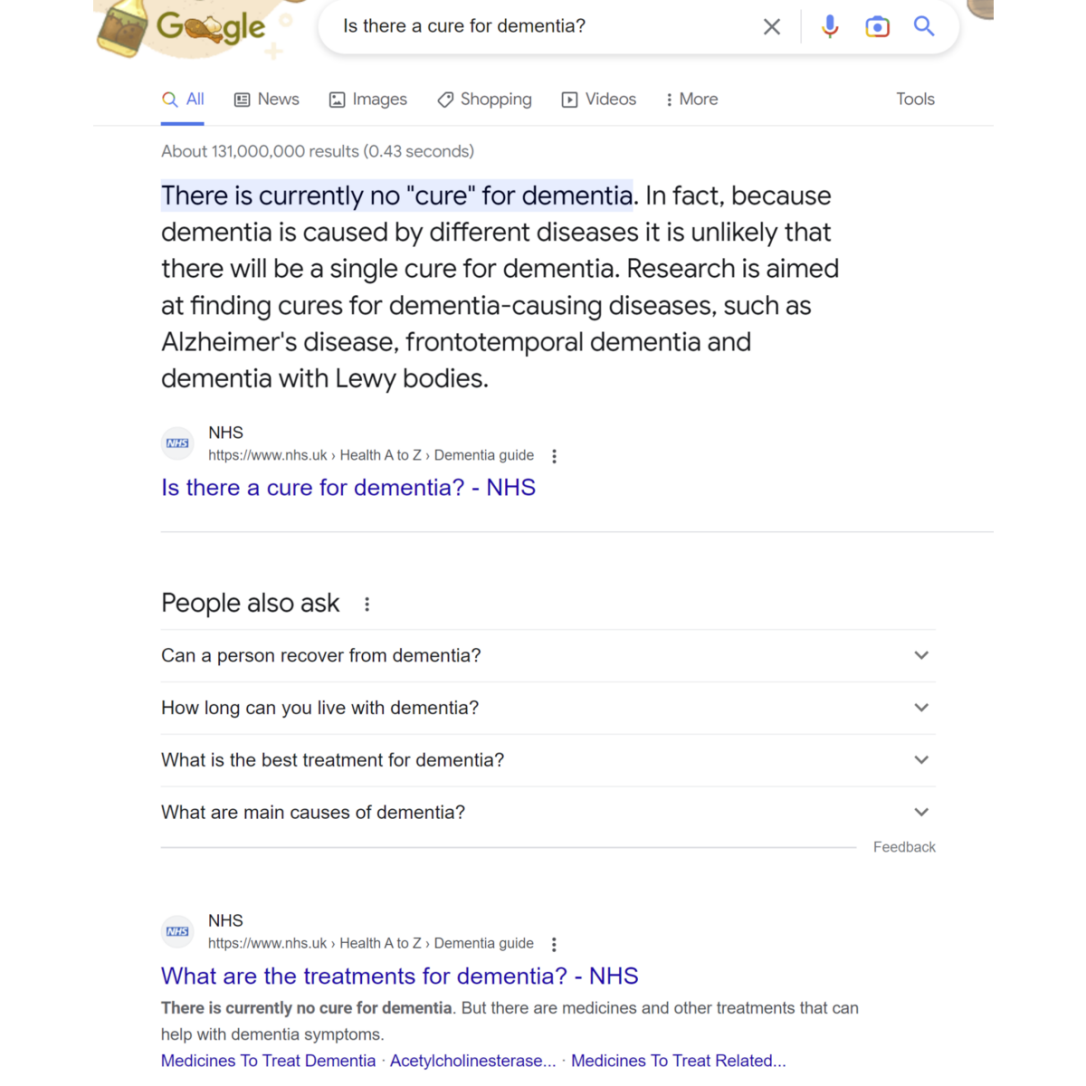
The answer displayed at the top of the results page on the Google search engine.

AI chatbots, such as ChatGPT, have accelerated the transition from keyword queries to question answering (QA) [8], where the goal is not to return a list of relevant pages but to answer the user’s question. Web search engines, such as Google, have also been slowly moving toward this direction. In particular, for some queries, the results page contains a short text snippet at the top of the page (also called answer box or quick answer or direct answer), as shown in Figure 1.

### Review of Query Tools

Web search engines, such as Google and Bing, continuously collect (crawl) content (documents) from the web, store it locally, and index it for faster retrieval at query time. When a user submits a query, the search engine uses a ranking algorithm to assign a score to each document and generate a ranked list of results [9]. Recently, search engines have been trying to move from *keyword search*, where a user provides keywords and the search engine returns a list of pages, to *QA* [8], where the user asks a question (eg, “What is the most popular drug for depression?”) and the search engine returns the answer (eg, “selective serotonin reuptake inhibitors [SSRIs]”) on the top of the results page, followed by a classic list of pages.

The exact ranking formula used by web search engines is not public, but the general technologies used are known. Specifically, before 2018, ranking used a combination of numerous features, such as the number of times query keywords appear in a document, the incoming hyperlinks of the document, and the importance of the domain. These features are combined using a function learned through machine learning [9].

A breakthrough came in 2018, when Google published a paper on bidirectional encoder representations from transformers (BERT) [10], which is a deep learning model for text. Specifically, BERT creates a semantic model of a text segment (a word, sentence, or paragraph), which then facilitates a semantic comparison of this text to a query. Some of the key advantages of BERT over previous keyword-based methods are as follows: (1) Synonyms are considered, or more generally alternative ways to express the same meaning; (2) all the words in the text are considered instead of just focusing on the most important words, such as nouns; (3) the order of the words becomes important; and (4) the exact segment of a document that answers the query is returned instead of returning the whole document. BERT has significantly improved the quality of search results and has brought web search engines closer to the goal of QA.

ChatGPT is a conversational agent, or chatbot, that can answer user questions in a way similar to how a human would respond [11]. ChatGPT is built on top of the third-generation Generative Pre-trained Transformer (GPT-3), which is a *language model* that has been trained using about 500 billion tokens (terms or words) [12]. Most of these tokens come from web pages (a subset of the pages crawled by a web search engine, such as Google), Wikipedia, and books. Importantly, language models are expensive to train, and hence, they are trained infrequently (eg, once a year), which means they may not contain the most current information available.

A language model can be viewed as a tool to generate reasonable continuations for a text segment. For example, for the text “it is sunny in,” a language model may suggest continuing this text with “California.” The key improvement of ChatGPT over GPT-3 is that it tries to make the chatbot responses useful to the user; that is, the response should answer the user’s question instead of just responding with some text that would naturally follow the user’s text in a document. To achieve that, ChatGPT is trained (more accurately, fine-tuned in addition to the training that GPT-3 already has) using human feedback to become more useful than GPT-3 [13]. Specifically, ChatGPT is fine-tuned using a technology called *reinforcement learning from human feedback*, whereby it can refine its subsequent responses based on the perceived usefulness of the answer to the current user.

ChatGPT only periodically gets retrained, every several months or even years, because of the high cost of training; that is, the response of ChatGPT is not up to date if it refers to recent events.

Access to ChatGPT is available on the ChatGPT website [14]. New users need to sign up using their email. They are provided with a small number of credits, and then they must pay a per usage fee computed based on the number of tokens submitted to ChatGPT.

### Potential of ChatGPT for Health Literacy

Although Google has been available for health information seeking for more than 2 decades, ChatGPT is a new resource that has the potential for promoting health literacy. However, given its recent availability to the public, there is limited research on how this technology may have practical applications in health care, especially for patient or caregiver education. Recently, Lee et al [15] suggested that ChatGPT has the potential to meet the needs of medical professionals related to medical note taking, answering medical problems for patient cases, and medical consultation [15]. In fact, the authors noted that when given a battery of medical test questions, ChatGPT provided correct responses 90% of the time. However, the information needs of patient and caregiver populations are quite different than those of trained medical professionals. For example, ChatGPT is not programmed to generate images, such as diagrams or other graphic tools, that a layperson may find helpful in understanding their health condition. However, an added feature of ChatGPT is that it allows users to easily change the style of a response to better match their profiles [13]. For example, one may ask, “Explain dementia to a 10-year-old child” or “Explain COVID-19 in a funny way” or “How would Obama explain a health care deductible?” To answer such questions, ChatGPT uses deep learning to paraphrase the responses, given the conversational model of the requested style.

There is no previous study analyzing the usefulness of ChatGPT for PLWD. This paper studied the potential of ChatGPT for health information seeking by PLWD. We investigated how ChatGPT compares to a web search for various types of query needs and presented our findings related to the strengths and weaknesses of each technology. We considered criteria such as reliability, accuracy, readability, and objectivity, which are critical for PLWD in evaluating health-related information.

## Methods

### Study Design

We developed a systematic strategy for data collection and analysis. This included identifying questions that caregivers commonly ask about Alzheimer disease and related dementias (ADRD) and caregiving, as well as establishing a set of criteria to assess the collected responses from ChatGPT and Google. More details on this process are outlined later.

### Question Identification

We established 2 categories of questions that caregivers commonly ask, informational and transactional questions (ie, questions about service delivery), which followed a common classification, as discussed earlier [7]. We considered 2 of the 3 categories of web questions for the reasons discussed in the *Introduction* section.

For general information questions, we modified individual items from the Alzheimer’s Disease Knowledge Scale (ADKS) [16]. The ADKS is a validated assessment tool that includes 30 statements about Alzheimer disease, and the participant selects *true* or *false* for each item*.* The statements address a number of topics related to assessment and diagnosis, caregiving, course, life impact, treatment and management, and symptoms. For example, consider the following 2 statements:

A person with Alzheimer disease becomes increasingly likely to fall down as the disease gets worse.

Having high blood pressure may increase a person’s risk of developing Alzheimer disease.

To convert these statements to questions, we added a prefix of “Is it true that” to each—for example, “Is it true that a person with Alzheimer disease becomes increasingly likely to fall down as the disease gets worse?” The ADKS is 1 of the most used tools to assess overall Alzheimer disease–related knowledge. These knowledge items across multiple domains were developed to identify gaps in patient and caregiver knowledge, specifically for those with dementia-related concerns seeking a dementia evaluation [16], making the tool appropriate for those searching for information.

The 30 transactional items used in this study represent commonly used dementia-related services and support. Families and caregivers confronted with caring for a loved one with dementia will often need to seek information about how to identify trustworthy, quality, local, and affordable services; these 30 items represent a spectrum of dementia-related services. Specifically, we considered questions related to how PLWD look for services they need within their local community [17]. We narrowed the list to 6 common categories of services: *adult day care*, *home health care*, *hospice care*, *respite care*, *memory clinics*, and *nonemergency medical transportation (NEMT)*. For each of these services, we created questions about *quality*, *accessibility*, and *affordability.* Specifically, we generated 5 questions for each service type. For example, here is a list of the 5 questions generated for *adult day care*:

- How do I pick the best adult day care? (Quality)
- How do I find adult day care in Riverside, California? (Accessibility)
- How do I pay for adult day care in California? (Affordability)
- How much does adult day care cost per day in Riverside, California? (Affordability)
- Does California license adult day care? (Quality)

The same questions were generated for each of the remaining 5 service types.

We used Riverside, California, as the location for our queries. Hence, we had a total of 30 transactional questions. The list of all 60 questions is shown in Multimedia Appendix 1.

### Data Collection and Management

**Figure 2:**
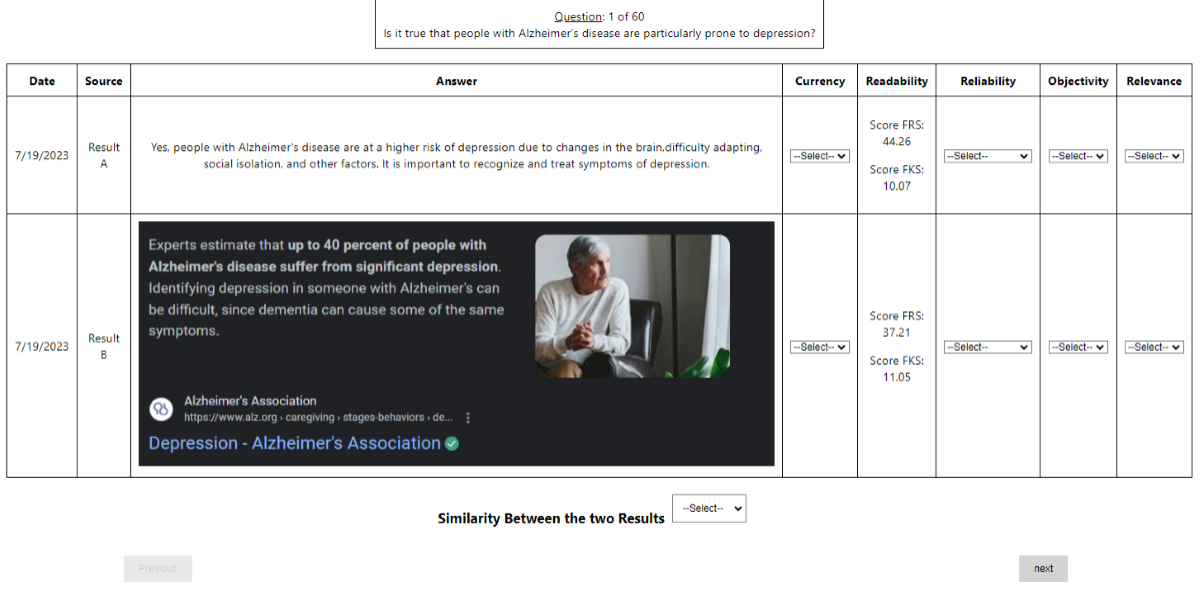
Screenshot of the evaluation web application. Each rater selects a response from the drop-down menus and then advances to the next question.

One member of the research team (author SG) identified responses by entering each question into the ChatGPT and Google search engines between March 10 and 13, 2023, using a computer located in Riverside, California. Another team member (author VH) supervised SG and reviewed the collection process. All team members provided feedback on the process. Next, a database was created that displayed the questions, each response, and the assessment criteria. The displaying of ChatGPT responses is straightforward, as each response is in plain text (see Figure 2). For Google, we ignored advertisement results and showed the answer box generated (direct answer, as shown in Figure 2) if any existed, or else we showed the top organic (ie, nonadvertisement) result (title and snippet). Due to the different formatting (plain text for ChatGPT and rich text for Google), it was not possible to mask the source, but the raters were not told the name of the platforms and were asked to disregard this factor in their assessments.

### Assessment Criteria

The rating system used was an extension of a rating system previously used to assess the content quality of dementia websites [18]. The 5 rating criteria we used are:

- Currency (yes, no, not sure): This criterion assessed whether information was provided on when the content was created and whether it was created within the past 5 years. This included the date of creation, the last updated date, and information in the actual response (eg, “In 2021, it was found that…”). Responses were deemed not current if they either lacked information about when the content was created or a date was provided but it was more than 5 years old. An assessment of “not sure” included cases where the response included text such as “Over the past year, it has been found that…,” where information about the date of creation was provided but we could not assess when it was published.
- Readability: The information should be clear and easy to understand by users of different literacy levels. To assess readability, we computed the Flesch Reading Ease Score (FRS) and the Flesch–Kincaid Grade Level Score (FKS) for each response [19]. The FRS ranges from 1 to 100, where 100 is the highest level of readability. A score of 60 is considered standard for publications targeting a general audience, and a score of 70 or more is considered easy for the average adult to read [20]. The FKS is calculated by translating the FRS into a grade reading level. For example, a score between 70 and 80 would translate to about an 8th-grade reading level.
- Reliability (reliable source, other source, no source, not sure): A response was deemed reliable if it provided the source of the content and the source was considered a reputable source for information about dementia, such as government websites and well-known organizations that provide information about dementia and caregiving (eg, Alzheimer Association). “Other source” referred to responses where a source was provided with the response (eg, website, organizational name) but it was not clear whether it was a reliable source. “No source” referred to responses that did not provide any information about the source. “Not sure” was used in cases where the response referred the reader to a reliable source for further information but there was no information about where the response content came from—for example, a response that read “Contact the Centers for Medicare and Medicaid for additional information” without offering information about the source of the response content.
- Objectivity (yes, no, not sure): A rating of “yes” reflected factual information that lacked any feelings or opinion about the content. “No” and “not sure” were assigned to cases where the content came from a for-profit agency (eg, Luxe Homecare) or if it was unclear whether the source was a for-profit organization.
- Relevance (high, medium, low, not sure): The raters evaluated the extent to which each response addressed the question asked. We also asked the raters to assess the similarity between the 2 results as “high,” “medium,” “low,” or “not sure.”

### Assessment Process

Initially, a reviewer-training session was conducted with 3 of the authors (EB, NR, and SS). Differences in rater responses were discussed to ensure uniform application of assessment criteria [21]. During the training, the authors rated the first 15 questions together, that is, they jointly assigned a single rating. The remaining 45 questions were then rated independently by each rater.

### Data Analysis

Frequencies and other descriptive statistics for all responses were generated. The Levene test was performed to assess equality in the variances between ChatGPT and Google readability scores on the FRS and FKS. This was followed by a 2-tailed Welch *t* test for independent samples to determine differences in the means of both groups. We computed the Fleiss κ to measure the agreement among the raters for the 45 questions that were rated independently.

## Results

For reproducibility purposes, we saved the results of our queries on ChatGPT and Google using the 60 questions [22].

### Currency

When comparing the responses as they related to the currency of information, we found that for ChatGPT, only 1 (1.7%) response listed the date when the data presented were collected, and the remaining 59 (98.3%) responses provided no dates. For Google, 19 (31.7%) responses provided dates when the data presented were collected or the dates when the source was updated, and dates fell within the past 5 years. The remaining 41 (68.3%) responses did not include dates when the information was provided or the dates provided were more than 5 years old. When comparing Google responses deemed current, there was no difference between informational (n=9, 30%) and transactional (n=10, 33.3%) questions. Table 1 illustrates the currency of both informational and transactional question responses.

**Table 1:** Currency for informational and transactional questions.

Currency	Transactional questions		Informational questions	
	ChatGPT, n (%)	Google, n (%)	ChatGPT, n (%)	Google, n (%)
Yes	0	10 (33.3)	0	9 (30.0)
No	30 (100.0)	20 (66.7)	30 (100.0)	21 (70.0)

### Reliability of Information

Regarding reliability, ChatGPT did not identify any sources for the information it provided in its responses. However, 4 (6.7%) responses were rated as *not sure* because they did not cite any sources but directed the user to contact an agency deemed reputable. For example, for the question “How do I find home health care in Riverside, California?” ChatGPT instructed the reader to contact the Riverside County Office on Aging. In Google, all responses provided the websites from where the information was sourced. Among these sources, 36 (60%) were deemed reliable (eg, Alzheimer Association, National Council on Aging) and 24 (40%) were unfamiliar to the reviewers.

Table 2 depicts the results of our analysis of the reliability of both informational and transactional questions. Reliability, in this context, refers to the extent to which the information provided by the source can be trusted as accurate and credible. The table shows that there was little difference between the *reliable source* and *no source* responses for both transactional and informational questions. However, for informational questions, the *other source* and *not applicable* responses were noticeably higher compared to transactional questions.

Regarding currency and reliability, we asked ChatGPT a follow-up question (“Where did you get this information?”), and it is worth noting that in almost all cases, ChatGPT responded that it uses a wide range of sources to generate responses, with no further details.

**Table 2:** Reliability for informational and transactional questions.

Reliability	Transactional questions		Informational questions	
	ChatGPT, n (%)	Google, n (%)	ChatGPT, n (%)	Google, n (%)
No source	28 (93.3)	0	30 (100.0%)	0
Not sure	2 (6.7)	0	0	0
Reliable source	0	16 (53.3)	0	25 (83.3)
Other source	0	14 (46.7)	0	5 (16.7)

### Objectivity

All the responses provided by ChatGPT were rated as objective, while 49 (81.7%) of the Google responses were deemed objective. When analyzed further, ChatGPT and Google performed similarly with regard to objectivity for informational questions. However, for transactional questions, 2 (3.3%) responses by Google were assessed as not being objective, because the source was a for-profit organization that provided services for PLWD, and it was unclear whether 11 (18.3%) of the sources were for-profit service agencies. Although the responses did not directly advertise services of the organization, the actual or potential conflicts of interest posed by the sources raised questions about the extent to which the response would be fully objective—for example, if a response to the query “How do I pick the best home health care?” was published on a website for an agency that provides home health care services.

Table 3 demonstrates the objectivity of the 2 types of questions used in this study. For informative questions, the responses were often more congruent, but they varied for transactional questions.

**Table 3:** Objectivity for informational and transactional questions.

Objectivity	Transactional questions			Informational questions	
	ChatGPT, n (%)	Google, n (%)	ChatGPT, n (%)		Google, n (%)
Yes	30 (100.0)	24 (82.7)	30 (100.0)		30 (100.0)
No	0	1 (3.5)	0		0
Not sure	0	4 (13.8)	0		0

### Relevance

For ChatGPT, reviewers rated 58 (96.7%) responses as being *highly relevant* to the question asked, 2 (3.3%) were rated as having *medium relevance*, and none of the responses were assessed as having *low relevance.* For Google, reviewers rated 36 (60%) responses as being *highly relevant* to the question, 4 (6.7%) were rated as having *medium relevance*, and 20 (33.3%) were assessed as having *low relevance.* Table 4 shows the results broken down by type of question. A comparison showed that the relevance of responses by ChatGPT was similar for both types of questions, whereas Google responses were more likely to be rated as having *medium* (n=5, 8.3%) or *low* (n=16, 53%) relevance for transactional questions. For transactional questions, the relevance of Google responses was typically ranked lower because Google drew upon sources that were referral services for dementia care or service providers themselves.

**Table 4:** Relevance for informational and transactional questions.

Relevance	Transactional questions			Informational questions	
	ChatGPT, n (%)	Google, n (%)	ChatGPT, n (%)		Google, n (%)
High	30 (100.0)	22 (73.3)	28 (93.3)		23 (76.7)
Low	0	7 (23.3)	2 (6.7)		1 (3.3)
Medium	0	1 (3.3)	0		6 (20.0)

### Similarity

Overall, the similarity between the content of ChatGPT and Google responses was rated as *high* for 13 (21.7%) responses, *medium* for 16 (26.7%) responses, and *low* for 31 (51.6%) responses. Our study found that the search results for informational questions were more similar than those for transactional questions, as shown in Table 5. These findings suggest that different types of queries may require different search strategies.

**Table 5:** Similarity for informational and transactional questions.

Similarity	Transactional questions, n (%)	Informational questions, n (%)
High	9 (32.1)	10 (33.3)
Medium	7 (25.0)	8 (26.7)
Low	12 (42.9)	12 (40.0)

### Readability

The readability of responses for both ChatGPT and Google varied widely, as displayed in Table 6. For ChatGPT, no responses had FRS ≥60 (indicating general reading audiences), while for Google, 12 (20%) responses had FRS ≥60. This was also the case for the FKS, where ChatGPT had 3 (5%) scores and Google had 21 (35%) scores at an 8th grade readability level or lower. However, there was greater variability in the readability scores of Google responses. The Levene test showed statistically significant differences in the variances between the ChatGPT and Google responses for the FRS (*F*_2,118_=11.16, *P*=.001) and the FKS (*F*_2,118_=9.89, *P*=.002). Therefore, a subsequent 2-tailed Welch *t* test for independent samples was performed, which also found statistically significant differences in the FRS (t_100_=–3.26, *P*=.001) and the FKS (t_100_=4.44, *P*<.001). Overall, the responses provided by Google had easier readability for general audiences, with a mean FKS of 9.86 (SD 3.47), which indicated that, on average, the Google responses were written at a 9th-grade reading level compared to ChatGPT, which had a mean FKS of 12.17 (SD 1.94), or a 12th-grade reading level.

The findings of this study are summarized in Table 7.

**Table 6:** Summary of FRS^a^ and FKS^b^ readability scores.

Score and variables		ChatGPT (N=60)		Google (N=60)
**FRS**				
	Mean (SD)	33.5 (12.46)	43.36 (19.61)	
	Variance	157.82	390.93	
	Range	4.84-57.81	4.11-91.84	
**FKS**				
	Mean (SD)	12.17 (1.94)	9.86 (3.47)	
	Variance	3.75	12.05	
	Range	7.48-16.11	2.71-19.39	

^a^FRS: Flesch Reading Ease Score.

^b^FKS: Flesch–Kincaid Grade Level Score.

**Table 7:** Summary of response assessment for ChatGPT and Google.

Assessment criterion	Superior performance	Comments
Currency	Google	No dates provided in ChatGPT results
Reliability	Google	No sources provided by ChatGPT
Objectivity	ChatGPT	Google more likely to generate responses from for-profit service agencies
Relevance	ChatGPT	Google’s relevance lower for informational queries
Readability	Google	Both ChatGPT and Google requiring relatively high-grade-level preparation

### Interrater Agreement

We calculated the Fleiss κ to determine whether there was agreement between the 3 raters in their assessments of currency, reliability, objectivity, and relevance. Overall, there was good reliability of the ratings of reliability (κ=0.732, 95% CI 0.655-0.808, *P*<0.001), currency (κ=0.731, 95% CI 0.614-0.848, *P*<.001), and relevance (κ=0.618, 95% CI 0.518-0.718, *P*<.001). In addition, there was fair agreement among the raters’ judgment of objectivity (κ=0.218, 95% CI 0.115-0.320, *P*<.001). The reason is that raters may have considered different ways for judging objectivity, including whether the source website was from a for-profit company, the content of the result, or even its tone.

## Discussion

### Principal Findings

The primary finding of this study is that ChatGPT and Google have complementary strengths for people with cognitive decline or their caregivers (Table 7). Specifically, ChatGPT provided more relevant responses to the queries (Google did not have an answer box in 8 of the 30 informational and 12 of the 30 transactional questions) and greater objectivity, given that Google was more likely to generate responses from sources that may have potential or actual financial conflicts of interest. Google has slowly been moving from keyword search to QA (see the *Review of Query Tools* section), which means that an increasing number of queries return answer boxes over time. Google has superior currency, as Google crawls web pages continuously to collect the latest content. However, ChatGPT only periodically gets retrained, as mentioned in the *Review of Query Tools* section. Further, both suffer from a lack of time stamps indicating how current a result is. Reliability is also a stronger point for Google, as it shows the sources (URLs) of each result in contrast to ChatGPT.

Reliability is critical, given that health misinformation on the internet has been shown to be a significant challenge for PLWD [23]. In some cases, perceptions of misinformation and scams result in some PLWD altogether avoiding searching for information about health-related topics on the internet [24]. PLWD have also reported frustration in reading about emerging research findings related to ADRD on the internet, only to find later that those findings were inaccurate as newer research is reported [23]. The impact of health misinformation on the internet on the dementia community has most recently been documented during the COVID-19 pandemic, where caregivers reported that they refused vaccination for themselves or their care recipients based on fears from what they read on the internet [25].

In addition to the currency and reliability limitations, we also observed a few other shortcomings of ChatGPT. First, it has limited support for other *languages* as its model has not been trained to the same level as in English. Second, it often *crashes* in the middle of a conversation or is unavailable (we expect this to improve over time). Third, it requires a monthly *subscription* to use (after a number of free accounts were given out), which is slowly changing as it is becoming part of the Microsoft Bing platform. Fourth, it is not able to help PLWD complete any *transactions*, such as administering a questionnaire to evaluate cognitive function or to find a doctor in their area. Fifth, when it does not fully understand a question, it does not ask any *clarification* question (eg, “Did you mean home care services?”). Instead, it responds as best it can based on the last input.

In this study, in terms of readability, ChatGPT responses tended to be too high in reading difficulty for a general audience, with readability ratings at an average 12th-grade reading level compared to Google’s average of a 9th-grade reading level. However, many of the Google responses were also more difficult to read than what is recommended for health education materials. The raters in this study were health and social service professionals with advanced education and training. Hence, their understanding of the ChatGPT responses may be higher compared to many PLWD. Health literacy, and more specifically digital health literacy, has been an ongoing challenge in the United States and is a priority for *Healthy People 2030*, which provides 10-year, measurable public health objectives and tools to help track progress toward achieving them [26].

Moreover, there are significant disparities in health and digital health literacy [26], which further exacerbates health inequalities for underserved populations of PLWD. Digital literacy has been identified as an ongoing challenge for PLWD, which was made more evident during the COVID-19 pandemic, when many caregivers needed to access digital platforms for health and support services [27]. Although the need for technology use during the pandemic may have increased digital literacy among this population, findings on this have not yet been reported. Prior research has found that there is a strong relationship between digital health literacy and overall health literacy for caregivers, which suggests that improving digital health literacy can improve outcomes such as self-efficacy for this population [28].

Although this analysis focused on the readability level of the responses generated, it also highlighted that many caregivers may have limits regarding health and computer literacy that impede their search strategies, such as the use of keywords. For instance, although the ChatGPT responses were deemed highly relevant to the questions posed in this study, caregivers with similar information needs may not generate the same responses due to less efficient queries.

The website of ChatGPT lists a few more limitations [11], which we include here for completeness:

(i) ChatGPT sometimes writes plausible-sounding but incorrect or nonsensical answers. (ii) ChatGPT is sensitive to tweaks to the input phrasing or attempting the same prompt multiple times. (iii) The model is often excessively verbose and overuses certain phrases. (iv) While we’ve made efforts to make the model refuse inappropriate requests, it will sometimes respond to harmful instructions or exhibit biased behavior.

### Ethical Issues

There are a number of ethical issues to consider regarding the use of ChatGPT and Google when searching for health information on the internet. For ChatGPT, 1 concern is that since the platform is trained on existing content on the internet, it may provide users with information that is inaccurate or highly biased [29]. User privacy has also been raised as an issue regarding AI browsing tools in general, with concerns about how user interactions with such technologies may be tracked [29]. Such ethical concerns have led to calls for a code of ethics for ChatGPT and similar AI tools [30]. Similarly, ethical issues regarding the use of Google for searching for health information have also been raised. For example, Google has been criticized for publicizing inaccurate public health data [31], concerns about privacy of user input [32], and biased algorithms that inform the top results of any given search query [33]. Hence, more work is needed on addressing ethical concerns in digital health literacy in general.

### Complementary Nature

Although ChatGPT can provide health-related information with high levels of accuracy [15], it has several drawbacks. For example, due to the lack of information about the sources of responses, users may need to cross-reference the responses by consulting with additional sources. This may increase the burden of information seeking [15]. Therefore, 1 potential result is that PLWD may find themselves using ChatGPT and Google in tandem rather than relying on one source or the other.

### Future Predictions

We expect that chatbot platforms, such as ChatGPT, and web search engines, such as Google, will slowly start to converge in the next few years. Specifically, chatbots will support timely results, show sources (eg, web page addresses), and offer multimedia (ie, images and videos) user interfaces. However, web search engines will increasingly move away from lists of pages to answers as results. For example, the results page (Figure 1) will increasingly emphasize the top answer (answer box), which will become more accurate, and downplay the subsequent list of web pages. Web search engines will also get better at exploiting the search (or conversational) history of the user.

### Related Work

#### Current State of Dementia-Focused Chatbots

Previously, we conducted a systematic review of commercially available chatbot apps that targeted PLWD [6]. Overall, we found that few chatbots focus on dementia and most are designed for engaging with PLWD, such as guided reminiscing. All but 1 chatbot were Alexa (Amazon) skill apps, which limits the target audience to those with Alexa-enabled technologies. However, research literature is increasingly focusing on this topic. For example, Varshini et al [34] recently reported the development of a *companion chatbot* with several safety and supportive features, such as communicating with family members about the location of PLWD and providing care recipients with reminders for memory support. Jiménez et al [35] presented a model for using Alexa for caregiver support when the care recipient presents problematic behaviors. However, the application of chatbot technologies for dementia-related health education is underdeveloped.

Recently, we completed a pilot test of an educational and supportive app for caregivers, called CareHeroes, that is multifunctional and includes an educational chatbot [36]. Other educational and supportive tools that are available on the app include links to vetted websites from trusted sources for dementia and caregiving, educational videos developed by the team and offered in English and Spanish, self-assessments for burden and depression that provide feedback based on the responses, and clinical assessments of care recipients. The chatbot is programmed to respond to common questions related to dementia and caregiving, and responses are based on content from the book *The Dementia Caregiver*, by Dr Marc E Agronin, a geriatric psychiatrist [37]. In the study, caregivers were asked to use the app for a period of 12 months and the authors tracked usage data from CareHeroes. Overall, they found that the educational chatbot is the most used educational feature on the app, with caregivers using it to gather information about depression and sleep problems experienced by the patients they cared for and about living wills. The findings suggest that chatbot technologies offer an opportunity to provide targeted education content to caregivers based on their individual informational needs. However, more research needs to be conducted to advance work in this area.

#### Searching the Web for Health Information

Caregivers have been demonstrated to be more active than noncaregivers in seeking information about health-related topics and often use the web to gather information from websites and social media [1]. However, caregivers’ experiences with using the web to gather information about health is not universal. For instance, a recent study analyzing data from the Health Information National Trends Survey (HINTS) found that 42.7% of caregivers in the United States do not have broadband access and there are significant disparities in access to broadband internet among caregivers [38]. This may limit access to web-based tools, such as ChatGPT and Google, especially for caregivers who may already be underserved. It has also been reported that caregivers from underserved populations, especially those who are immigrants, have more difficulty in finding the information they are seeking from web searches and are less likely to trust the information that they find [1]. To support caregivers in the future in health-related web searching, policy and research should focus more on advancing infrastructure for high-speed internet access and increasing digital health literacy among caregivers.

#### ChatGPT in Health Care

Research on using ChatGPT in health care apps is still in its infancy, although research on its potential use is increasing. A recent systematic review found that a number of potential benefits of using ChatGPT in health care settings have been identified in the literature, including improving health care services and health literacy, supporting research, and educating the health care workforce [30]. However, the same review identified a number of concerns with ChatGPT in health care apps, including ethical concerns around potential bias, the risk of spreading misinformation, and the security of protected health information. The literature on ChatGPT in health care has mostly focused on providers, educators, and researchers. Little has been done to explore its use for patient education. In their study using ChatGPT to answer questions about prostate cancer, Zhu et al [39] found that although all the large language models (LLMs) they submitted their questions to provided more than 90% accuracy, ChatGPT had the highest accuracy rate. They also noted that the free version of ChatGPT performs better than the paid version. However, they did not compare the responses from any of the LLMs with those from more traditional search engines, such as Google, as we set out to do. In addition, their study was similar to ours, in that they generated questions to ask the chatbots rather than examining how real PLWD interact with these search tools. Therefore, more studies are needed to better understand how ChatGPT and similar models can be appropriately used to improve health literacy.

### Study Limitations

Although there were numerous considerations made to increase the rigor of this study, there are some limitations. First, the study was conducted in the midst of evolving AI technology; therefore, these findings are snapshots in time. Second, the service-related queries focused on a specific urban area, so we anticipate that these findings would be similar in other locations. Third, although we tried to identify frequently asked questions, the queries did not consider slang or cultural idioms and all queries were in English. Finally, the raters could guess which results corresponded to Google and which to ChatGPT, based on their presentation, as shown in Figure 2.

### Conclusion

This paper studied how Google and ChatGPT compare in answering queries related to dementia or other cognitive decline. In total, 60 informational and transactional queries were selected, and their results were rated by 3 experts based on several criteria. We found that both Google and ChatGPT have strengths and weaknesses. Google more often provides the source and date of the response, whereas ChatGPT has a higher response accuracy and objectivity. Their combination could potentially provide results of higher quality. That is, more research and new technologies are needed that will leverage the language understanding and precision of ChatGPT and combine it with the wide coverage and currency of Google.

## Appendix

Multimedia Appendix 1 List of questions.

## Abbreviations

AI: artificial intelligence
ADKS: Alzheimer’s Disease Knowledge Scale
ADRD: Alzheimer disease and related dementias
BERT: bidirectional encoder representations from transformers
FKS: Flesch–Kincaid Grade Level Score
FRS: Flesch Reading Ease Score
GPT-3: third-generation Generative Pre-trained Transformer
LLM: large language model
PLWD: people living with dementia or other cognitive decline and their caregivers
QA: question answering

## Data Availability

The data sets generated and analyzed during this study are available in Multimedia Appendix 1 and in a repository [22].
