# Supplementary material for: ChatGPT vs Google for Queries Related to Dementia and Other Cognitive Decline: Comparison of Results
Source: J Med Internet Res. 2023 Jul 25;25:e48966. doi: 10.2196/48966 (PMC10410383; doi:10.2196/48966)
Supplement: Multimedia Appendix 1 [file jmir_v25i1e48966_app1.docx]

## List of questions

1. Is it true that people with Alzheimer disease are particularly prone to depression?
2. Is it false that it has been scientifically proven that mental exercise can prevent a person from getting Alzheimer disease?
3. Is it true that after symptoms of Alzheimer disease appear, the average life expectancy is 6-12 years?
4. Is it true that when a person with Alzheimer disease becomes agitated, a medical examination might reveal other health problems that caused the agitation?
5. Is it true that people with Alzheimer disease do best with simple instructions, given 1 step at a time?
6. Is it true that when people with Alzheimer disease begin to have difficulty in taking care of themselves, caregivers should take over right away?
7. Is it true that if a person with Alzheimer disease becomes alert and agitated at night, a good strategy is to try to make sure that they get plenty of physical activity during the day?
8. Is it true that in rare cases, people have recovered from Alzheimer disease?
9. Is it true that people whose Alzheimer disease is not yet severe can benefit from psychotherapy for depression and anxiety?
10. Is it true that if trouble with memory and confused thinking appears suddenly, it is likely due to Alzheimer disease?
11. Is it true that most people with Alzheimer disease live in nursing homes?
12. Is it true that poor nutrition can make the symptoms of Alzheimer disease worse?
13. Is it true that people in their thirties can get Alzheimer disease?
14. Is it true that a person with Alzheimer disease becomes increasingly likely to fall down as the disease gets worse?
15. Is it true that when people with Alzheimer disease repeat the same question or story several times, it is helpful to remind them that they are repeating themselves?
16. Is it true that once people develop Alzheimer disease, they are no longer capable of making informed decisions about their own care?
17. Is it true that eventually, a person with Alzheimer disease will need 24­hour supervision?
18. Is it true that having high cholesterol may increase a person’s risk of developing Alzheimer disease?
19. Is it true that tremor or shaking of the hands or arms is a common symptom in people with Alzheimer disease?
20. Is it true that symptoms of severe depression can be mistaken for symptoms of Alzheimer disease?
21. Is it true that Alzheimer disease is 1 type of dementia?
22. Is it true that trouble handling money or paying bills is a common early symptom of Alzheimer disease?
23. Is it true that 1 symptom that can occur with Alzheimer disease is believing that other people are stealing one’s things?
24. Is it true that when a person has Alzheimer disease, using reminder notes is a crutch that can contribute to decline?
25. Is it true that prescription drugs that prevent Alzheimer disease are available?
26. Is it true that having high blood pressure may increase a person’s risk of developing Alzheimer disease?
27. Is it true that genes can only partially account for the development of Alzheimer disease?
28. Is it true that it is safe for people with Alzheimer disease to drive as long as they have a companion in the car at all times?
29. Is it true that Alzheimer disease cannot be cured?
30. Is it true that most people with Alzheimer disease remember recent events better than things that happened in the past?
31. How do I pick the best adult day care?
32. How do I find adult day care in Riverside, California?
33. How do I pay for adult day care in California?
34. How much does adult day care cost per day in Riverside, California?
35. Does California license adult day care?
36. How do I pick the best home health care?
37. How do I find home health care in Riverside California?
38. How do I pay for home health care in California?
39. How much does home health care cost per day in Riverside, California?
40. Does California license home health care?
41. How do I pick the best hospice care?
42. How do I find hospice care in Riverside, California?
43. How do I pay for hospice care in California?
44. How much does hospice care cost per day in Riverside, California?
45. Does California license hospice care?
46. How do I pick the best respite care?
47. How do I find respite care in Riverside, California?
48. How do I pay for respite care in California?
49. How much does respite care cost per day in Riverside, California?
50. Does California license respite care?
51. How do I pick the best memory clinics?
52. How do I find memory clinics in Riverside, California?
53. How do I pay for memory clinics in California?
54. How much do memory clinics cost per day in Riverside, California?
55. Does California license memory clinics?
56. How do I pick the best nonemergency medical transportation?
57. How do I find nonemergency medical transportation in Riverside, California?
58. How do I pay for nonemergency medical transportation in California?
59. How much does nonemergency medical transportation cost per day in Riverside, California?
60. Does California license nonemergency medical transportation?
